# Automatic generation of pseudoknotted RNAs taxonomy

**DOI:** 10.1186/s12859-023-05362-5

**Published:** 2023-06-15

**Authors:** Michela Quadrini, Luca Tesei, Emanuela Merelli

**Affiliations:** School of Sciences and Technology, University of Camerino, Via Madonna delle Carceri 7, 62032 Camerino, MC Italy

**Keywords:** RNA secondary structures, Evaluation framework, Benchmark, RNA comparison methods, Agglomerative clustering

## Abstract

**Background:**

The ability to compare RNA secondary structures is important in understanding their biological function and for grouping similar organisms into families by looking at evolutionarily conserved sequences such as 16S rRNA. Most comparison methods and benchmarks in the literature focus on pseudoknot-free structures due to the difficulty of mapping pseudoknots in classical tree representations. Some approaches exist that permit to cluster pseudoknotted RNAs but there is not a general framework for evaluating their performance.

**Results:**

We introduce an evaluation framework based on a similarity/dissimilarity measure obtained by a comparison method and agglomerative clustering. Their combination automatically partition a set of molecules into groups. To illustrate the framework we define and make available a benchmark of pseudoknotted (16S and 23S) and pseudoknot-free (5S) rRNA secondary structures belonging to Archaea, Bacteria and Eukaryota. We also consider five different comparison methods from the literature that are able to manage pseudoknots. For each method we clusterize the molecules in the benchmark to obtain the taxa at the rank phylum according to the European Nucleotide Archive curated taxonomy. We compute appropriate metrics for each method and we compare their suitability to reconstruct the taxa.

## Background

RNA is a single-stranded polymer made of four types of nucleotides—Adenine (A), Guanine (G), Cytosine (C), and Uracil (U)—linked together by phosphodiester bonds. RNA can encode genetic information and perform biological functions in molecular processes including transcription, splicing, translation, and regulation of protein function. RNA folds on itself forming a three-dimensional shape by establishing hydrogen bonds mainly forming Watson-Crick (G-C and A-U) and wobble (G-U) base pairs. Such spatial configuration of an RNA is tied to its biological functions [1]. Knowledge of similarity between molecules permits to group them into families, infer their evolutionary history, detect functional motifs and, thus, predict their biological function [2].

Functional non-coding RNAs such as transfer RNA (tRNA) and ribosomal RNA (rRNA) that perform different but cooperative functions in protein synthesis exhibit a spatial configuration—i.e., a *structure*—that is highly conserved despite possible differences in the sequence of nucleotides (also called *primary sequence*) [3, 4]. In fact, while the sequence variations contribute to differences among species, the structure is necessary for cell function and is evolutionary conserved.

Biological classification started formally in 1758 with Linneus’s work [5]. The aim of this research area is to produce biological taxonomies in which one or more populations of an organism or organisms are grouped together due to similar characteristics. Such groups are called taxa and each defined taxon has a name and belongs to a taxonomy rank in the defined hierarchy. Taxonomy ranks that are typically used are: domain, kingdom, phylum (division in botany), class, order, family, genus and species. Since 1990, thanks to the work of Woese et al. [6], the general accepted classification of cellular life at the *domain* taxonomy rank is: *Archaea*, *Bacteria* and *Eukaryota*. A major breakthrough in the reconstruction of phylogenies, mainly regarding microbial life (which is the large majority of life on Earth), was the use of the 16S rRNA, a component of prokaryotic ribosomes, because it is present in all cells and shows a slow rate of evolution [7]. Current existing taxonomies are phylogenetic tree-guided and manually curated. They strongly rely on the molecular approach on 16S rRNA and other ribosomal RNAs like 23S and 5S. Different taxonomies are available in public databases, e.g. RDP-II [8], Greengenes [9] and SILVA [10, 11].

**Fig. 1:**
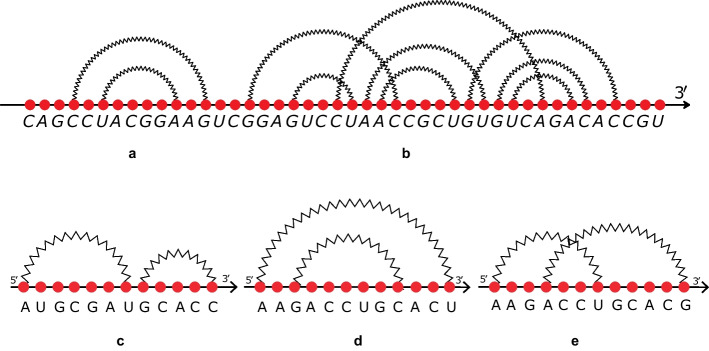
On the top, an RNA secondary structure illustrated via an arc-diagram. The motif in part **a** is pseudoknot-free, while the one in part **b** is pseudoknotted. Pseudoknots are clearly visible as crossings of arcs. On the bottom, the three feasible relations: **c** concatenation, **d** nesting and **e** crossing of two bonds

In the literature, most approaches to compare RNAs focus on the *secondary structure*, an abstraction of the spatial three-dimensional shape obtained disregarding the molecular form and considering only phosphodiester bonds between consecutive nucleotides (referred to as strong bonds) and hydrogen bonds (also called weak bonds). This abstraction represents an intermediate level between the primary sequence and the shape and has the advantage of being both relevant from a biological perspective and tractable from a computational point of view. An RNA secondary structure is said to be pseudoknot-free if its arc-diagram does not present crossings among weak bonds (Fig. 1a) and it is called pseudoknotted otherwise (Fig. 1b). The arc-diagram of an RNA secondary structure is a graph whose vertices, represented on a horizontal line, identify nucleotides, while the arcs that connect two non-consecutive vertices correspond to weak bonds. Comparison methods are typically based on the natural mapping between secondary structures without pseudoknots and trees. They apply algorithms based on tree edit or tree alignment to quantify the differences between the two molecules. An approach based on tree edit distance is implemented in the tool RNAdistance [12], while another using tree alignment is developed in RNAforester [3, 13]. Both are distributed with the Vienna RNA package [14]. Other techniques that consider different secondary structure representations, such as multilayer models or arc-annotated sequences, are also based on tree edit and tree alignment distance. Some implementations of these approaches are MiGaL [15], TreeMatching [16], Gardenia [17], and RNAStrAT [18].

Motifs with pseudoknots play a central role in cellular activities, but they are excluded from most of the comparison approaches in the literature because the classical tree representation of structures fails when these motifs are present. However, some alternative techniques have been defined to classify RNA structures with pseudoknots. A concept of shape was introduced by Giegerich et al. [19], and a topological classification based on a topological invariant, the *Genus*, was introduced by Bon et al. [20]. To simplify the computation, Reidys *et al. *introduced the concept of shadow of an arc-diagram [21]. To such a topological classification, Vernizzi *et al. *added a new topological invariant, the crossing number, associated to the shadow of the arc-diagram [22]. It is the number of crossings of the shadow arcs. These topological-based approaches permit the classification of pseudoknotted RNA molecules into families, but they cannot infer their evolutionary history or detect functional motifs. For example, Quadrini showed that a set of 16S rRNA, with or without the effects of drugs, are classified in the same group [23]. Moreover, their classification methods find groups that do not correspond exactly to the known taxonomies. Matsui *et al. *proposed a method that considers tree adjoining grammars for modeling pseudoknots and extended hidden Markov models on tree structures to pair stochastic tree adjoining grammars [24]. Chiu and Chen developed heuristics, called Progressive Stem Matching (PSM), to align a pair of RNA secondary structures with arbitrary pseudoknots by identifying conserved stems of the two RNA molecules [25]. A tool called PSMAlign was made available for computing the alignment. We introduced a tree grammar to model RNA secondary structures with arbitrary pseudoknots and defined the Algebraic Structural Pseudoknot RNA Alignment (ASPRA) distance to quantify the structural differences between pairs of molecules [26]. Furthermore, we developed the tool ASPRAlign to compute the ASPRA distance between two or more molecules [27]. The distance is obtained by aligning particular tree representations of RNAs. These trees neglect the primary sequence by considering only the structure of the molecules to be aligned. Wang et al. [28] proposed a tree representation of structures with pseudoknots by topological centroid identification and their comparison methods based on the tree edit distance. Antczak et al. [29] introduced a concept of Pseudoknot Order (PskOrder) that gives a measure of the pseudoknot complexity, which allows the connection to the hierarchy of RNA folding introduced by Zok et al. [30]. A direct application is the encoding of an RNA structure with pseudoknots into the dot-bracket-letter notation [31, 32]. RAG-2D (RNA As Graph) is an approach that permits to describe the topology of molecules using the Laplacian matrix of a graph. Schlick and co-workers defined *dual graphs* to formalize RNA secondary structures using connectivity information among the loops of the structures. Their approach permits to exploit concepts of graph theory to study the relationships between structure and function in RNAs also in case of pseudoknotted structures [33–35]. In particular, they defined two features, based on the Laplacian matrix, that reflect the graph topology of a structure. Using these features it is possible to define a distance between two structures [36].

To the best of our knowledge the works presented above are the up-to-date available studies in the long-standing problem of comparison of RNA secondary structures in presence of pseudoknots. Evaluating the performance of these techniques is certainly an important aspect, but unfortunately, as we mentioned above, a framework for evaluating approaches that can manage structures with pseudoknots is not available in the literature. BRASERO, a benchmark, was introduced by Allali et al. [37] in 2012 for comparìing the performance of tools that accepts only pseudoknot-free structures.

This work aims at defining an evaluation framework for comparison methods that manage pseudoknotted RNA structures. The framework assumes that it is possible to obtain a similarity/dissimilarity measure between any pair of structures from any considered comparison method. This measure is used as a basis for applying agglomerative clustering, one of the most common types among hierarchical clustering approaches [38], in order to compute a partition of a given set of molecules. To illustrate and test the framework, we select several sets of molecules and automatically partition them to reconstruct their taxa according to a certain taxonomic rank of a hierarchical taxonomy. The execution of the framework requires the selection of a manually curated biological taxonomy, for instance one of those that are present in SILVA [10, 11] or other databases, and select a taxonomy rank, e.g., phylum, class, order and so on. Then, agglomerative clustering is run on each set of molecules imposing a number of clusters equal to the number of taxa in the selected taxonomy rank. The precision of the computed partition w.r.t. the labels in the curated biological taxonomy is then computed using appropriate metrics.

More in detail, we define an open-data repository that consists of 504 molecules of pseudoknotted 16S rRNA, 68 molecules of pseudoknotted 23S rRNA and 174 pseudoknot-free molecules of 5 S rRNA belonging to the generally accepted three life domains Archaea, Bacteria, and Eukaryota. We then reconstruct their taxa at the *phylum* taxonomy rank according to the European Nucleotide Archive (ENA) taxonomy [39]. The repository of molecules is available at https://doi.org/10.6084/m9.figshare.20731783.v1. The scripts to execute the framework and the processed molecules are available at https://github.com/bdslab/TaxonClassifier. They can be used to reproduce all the results presented in this work and to try to reconstruct different curated taxonomies at different taxonomy ranks of the same dataset or other datasets. Moreover, they can be adapted to classify other kind of features of a set of molecules, such as their known function or their types of pseudoknots.

To show how the evaluation framework can be used, we apply it to five comparison methods: Genus, PSMAlign, ASPRAlign, PskOrder and RAG-2D. ASPRAlign was introduced by the same authors of this paper while the other methods were developed by other research groups. The dissimilarity measures provided by the methods are evaluated w.r.t. their suitability to classify the molecules in groups corresponding to their phylum. We compute all the performance scores using the rand index, homogeneity, and completeness scores metrics [40, 41]. The metrics are computed for each method, varying the parameters of the clustering algorithm. The computed metrics show that, in general, Genus and PskOrder have a lower performance than the other methods in reconstructing the phyla as they make a high abstraction on the information contained in the secondary structure. In the case of 16S structures, PSMAlign, ASPRAlign and RAG-2D show similar scores with performance varying slightly according to the chosen parameter of the clustering algorithm and the chosen metric for the clustering evaluation. Archaea are better reconstructed by ASPRAlign while, for Bacteria and Eukaryota, phyla are better reconstructed by PSMAlign in some cases and by ASPRAlign in some other cases. However, the scores of PSMAlign and ASPRAlign have slight differences, also w.r.t. the scores of RAG-2D. For Eukaryota, the best scores are lower than those of Archaea and Bacteria and this suggests a further analysis on lower taxonomy ranks, such as class or order. In the case of 23S structures, the results are similar to the 16S ones, but in this case the best scores of Eukaryota are not lower than the Archaea and Bacteria ones. This might suggest that for 23S the complexity of the organism does not affect significantly the reconstruction of the phyla. Finally, for the pseudoknot-free 5S structures we observe a significant predominance of PSMAlign, which confirms an already documented experiment in which the evaluation framework BRASERO was used [25].

## Results

We illustrate the definition of the framework, which is the main result of this paper. This includes the construction of the benchmark, which is a fundamental part of the evaluation framework, and how this is to be executed. Then, we also show the results of the computations that we obtain applying the framework to the five selected comparison methods.

### Construction and content of the benchmark

In this paper we use the evaluation framework for assessing the suitability of different comparison methods in reconstructing the phylogeny of sets of molecules. To this end we selected and processed a benchmark containing all the *pseudoknotted* 16S and 23S rRNA secondary structures that were available from the Comparative RNA Website (CRW) version 2 at https://crw-site.chemistry.gatech.edu/. This database consists of entries of ribosomal and intronic RNA molecules obtained by covariance-based comparative sequence analysis [42]. Our choice was mainly motivated by the fact that 16S and 23S are the molecules for which the taxonomy is better studied. Moreover, we selected all the available 5S rRNA secondary structures in order to have a control sub-set and to test the framework also on pseudoknot-free structures.

The three groups of selected molecules are formed by 174 entries of 5S, 504 entries of 16S and 68 entries of 23S. They show an increasing complexity at the level of pseudoknots. As complexity measure we consider the concept of PskOrder [29]. 5S molecules are the most simple structures in our benchmark as they are pseudoknot-free, i.e., with PskOrder equal to zero. All 16S molecules have PskOrder equal to 1, i.e., they are characterized by at most two arcs crossing each other. 23S molecules show a PskOrder in the range 2–4, mostly of value 3. This variability of complexity in the benchmark mitigates the presence of possible biases in the results of the evaluation due to the particular selection of molecules that is made. It must be said that the selection, in general, is limited by the availability of secondary structures with pseudoknots in public databases.

Statistical information on the benchmark is reported in Table 1. A few of the selected structures contained errors in the format or belonged to organisms that were not classified in the ENA taxonomy. In those cases the structures were excluded from the benchmark.

**Table 1:** Statistical information of the molecules in the benchmark

Life domain	Type	Number of entries	Medium of lenghts	Variance of lengths	Medium of pair numbers	Variance of pair numbers
Archaea	5-S	26	124	10	41	5
Bacteria	5-S	71	121	4	39	1
Eukaryota	5-S	77	120	5	37	0
Archaea	16-S	24	1485	180	465	29
Bacteria	16-S	200	588	588	469	60
Eukaryota	16-S	280	1576	117808	433	8298
Archaea	23-S	4	2953	1904	860	118
Bacteria	23-S	43	2914	3018	865	352
Eukaryota	23-S	21	2578	1314895	662	102216

#### Data processing

We created a unique and stable identifier for each entry in the benchmark. The ID provides some molecular information and permits the expansion of the benchmark by keeping all previous IDs unchanged. The molecular information that is provided, as spreadsheets, with the benchmark itself consists of the name of the organism to which the molecule belongs together with its phylogenetic information assigned by the ENA taxonomy [39]: the phylum, the class and the order. This choice does not prevent the use of other curated taxonomies and also other taxonomic ranks, for instance at lower level. Any taxonomy database can be searched, for instance, through the SILVA website https://www.arb-silva.de using the organism name for getting the relative taxa to be used in different executions of the framework.

One of the challenging tasks in the building of the benchmark arose from the fact that the comparison tools take various formats as input and sometimes they do not accept header information or comments. Therefore, we organized the benchmark into six folders, each containing a different format: BPSEQ, CT, dot-bracket-letter (db) and the same formats without header information or comments (BPSEQ-nH, CT-nH and db-nH). We initially collected the molecules in the BPSEQ and CT formats from the CRW website and we converted them into dot-bracket-letter notation using the RNApdbee 2.0 online tool [29, 32]. Then we processed all the files to eliminate headers and comments. In the files with the header, anyway, the original accession number of the molecule is available for searching in other databases. Inside each format folder the molecules are divided into the three domain groups: Archaea, Bacteria, and Eukaryota. Each group contains the 5S, 16S and 23S subfolders with the actual secondary structure files.

The molecules of the benchmark, together with the spreadsheets reporting the list of all the molecules together with their ENA taxonomy information can be freely accessed and used at https://doi.org/10.6084/m9.figshare.20731783.v1 under the CC-BY 4.0 License. The evaluation framework and its application to the molecules of the benchmark are available at https://github.com/bdslab/TaxonClassifier under the GNU General Public License v3.0.

### Execution of the framework

**Fig. 2:**
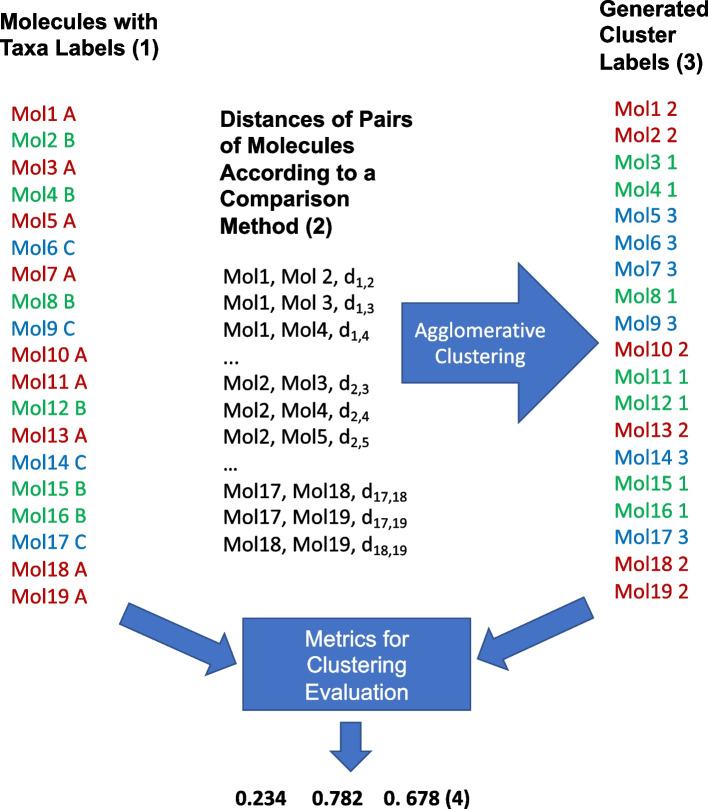
Execution of the evaluation framework on a set of molecules. Required input is: a list of the molecules with the corresponding taxa labels at a chosen taxonomy rank of a curated taxonomy (1); a CSV file with the distances between all pairs of molecules in the set, computed with a selected comparison method (2). Agglomerative clustering is applied using the given distances to generate a number of clusters equal to the number of different input taxa labels. The result is a list of molecules with assigned cluster labels (3). Metrics to evaluate how well the generated cluster labels match the original ones are computed and outputted (4)

Figure 2 shows how the evaluation framework is executed on a selected set of molecules that are assigned a known label corresponding to the taxa of a chosen taxonomy rank according to a curated biological taxonomy (Fig. 2(1)).

In our experiment, we selected the sets Archaea 16S, Archaea 23S, Archaea 5S, Bacteria 16S, Bacteria 23S, Bacteria 5S, Eukaryota 16S, Eukaryota 23S and Eukaryota 5S from our benchmark. Each element of the sets was associated with the corresponding phylum label from the ENA taxonomy. The folder RNAs/Molecules of the repository https://github.com/bdslab/TaxonClassifier contains a CSV files for all the sets in which the required input information is stored.

The molecules of the set must then be compared pairwise using a selected comparison method. A CSV file must be constructed from the computed dissimilarity or similarity measures containing the distance between any pair of the molecules in the set (Fig. 2(2)). Dissimilarity values can be directly interpreted as distances while, in case similarity values *sim* are made available by the comparison method, they can be easily converted into dissimilarity ones *diss* by normalizing them into the interval [0, 1] and considering $diss = 1 - sim$.

We executed the comparison for all the sets of our experiment and for all the five methods illustrated in “Methods” Section. The computed values could be directly interpreted as distances for Genus, PSMAlign, ASPRAlign and PskOrder. For RAG-2D we obtained a CSV file containing, for each molecule, a pair of features derived from the eigenvalues of the Laplacian matrix of the dual graph associated to the structure (see “Methods” Section). The distances among any pair of molecules are then derived from these features. The scripts that were used to execute all the methods are available in the folder RNAs/Computation of the repository https://github.com/bdslab/TaxonClassifier and can be freely reused to compute the comparison on other sets of molecules or can be adapted for other comparison methods. The computed distances or features for all the sets in our experiment are available in the folder RNAs/Distances of the same repository.

The CSV file containing the distances or the features is given as input to an agglomerative clustering algorithm that assigns labels to the elements of the set according to the computed clusters (Fig. 2(3)). We furnish a Python script executing this task using $\mathtt {scikit-learn}$, a free software machine learning library for the Python programming language [43, 44]. The script is available at https://github.com/bdslab/TaxonClassifier in two versions: ClusterMatrix.py, which processes CSV files containing distances, and ClusterFeatures.py, which processes CSV files containing pairs of features. The clusters are computed using the single, complete and average methods, as specified in “Methods” Section. The molecules are clusterized into a number of clusters equal to the number of taxa at the taxonomy rank of the chosen taxonomy. This parameter is automatically computed by the script. For instance, in our experiment, Archaea 16S are grouped into 3 clusters because in the ENA taxonomy the number of phyla of the molecules present in the benchmark are just three: Euryarchaeota, Crenarchaeota, and Nanoarchaeota.

Finally, the script computes and outputs the values of the three metrics rand index, homogeneity and completeness (see “Methods” Section) taking as input the original labels of the curated taxonomy and the labels computed by the clustering algorithm (Fig. 2(4)).

The results of the execution of the framework on our five comparison methods for the Archaea 16S, Bacteria 16S and Eukaryota 16S rRNAs are reported in Tables 2, 3 and  4 where simple, complete and average linkage was used as parameter of the clustering algorithm, respectively. Tables 5, 6 and 7 report the results for the 23S rRNAs following the same scheme. Finally, Tables 8, 9 and  10 contains the results for the 5S rRNAs, which are the pseudoknot-free ones. The same results are available as output of the scripts in the folder RNAs/Benchmark of the repository https://github.com/bdslab/TaxonClassifier. The outputs obtained in our experiment are illustrated and discussed in “Discussion” Section.

**Table 2:** Results of the evaluation framework applied to the 16S rRNA benchmark in which molecules are divided into Archaea, Bacteria and Eukaryota. Single linkage was used in clustering

	Genus	PSMAlign	ASPRAlign	PskOrder	RAG-2D
*Archaea*					
Rand index	0.514	0.594	**0.920**	0.601	0.547
Homogeneity	0.037	0.288	**0.833**	0.118	0.127
Completeness	0.073	0.427	**0.778**	0.230	0.099
*Bacteria*					
Rand index	0.407	0.495	0.461	0.302	**0.696**
Homogeneity	0.100	**0.357**	0.276	0.074	0.240
Completeness	0.206	**0.843**	0.564	0.279	0.215
*Eukaryota*					
Rand index	0.410	0.450	0.457	0.237	**0.611**
Homogeneity	0.110	**0.234**	0.231	0.065	0.218
Completeness	0.296	**0.591**	0.560	0.406	0.311

The highest score for each row is written in bold

The scores of the three metrics rand index, homogeneity and completeness are shown where the taxa were constructed basing on dissimilarity matrices taken from genus topological invariant (Genus), PSM (PSMAlign tool) ASPRA distance (ASPRAlign tool), Pseudoknot Order (PSkOrder) and RNA as Graph Fiedler Vector approach (RAG-2D)

**Table 3:** Results of the evaluation framework applied to the three life domains (Archaea, Bacteria and Eukaryota) of 16S rRNA benchmark where complete linkage was used in clustering

	Genus	PSMAlign	ASPRAlign	PskOrder	RAG-2D
*Archaea*					
Rand index	0.565	0.721	**0.746**	0.601	0.547
Homogeneity	0.134	**0.833**	**0.833**	0.118	0.128
Completeness	0.198	0.541	**0.559**	0.230	0.100
*Bacteria*					
Rand index	0.453	**0.847**	0.818	0.302	0.707
Homogeneity	0.146	**0.811**	0.684	0.074	0.253
Completeness	0.272	**0.631**	0.582	0.279	0.212
*Eukaryota*					
Rand index	0.450	0.734	0.768	0.248	**0.782**
Homogeneity	0.122	0.455	**0.474**	0.074	0.302
Completeness	0.295	0.476	**0.520**	0.413	0.272

The highest score for each row is written in bold

**Table 4:** Results of the evaluation framework applied to the three life domains (Archaea, Bacteria and Eukaryota) of 16S rRNA benchmark where average linkage was used in clustering

	Genus	PSMAlign	ASPRAlign	PskOrder	RAG-2D
*Archaea*					
Rand index	0.565	0.431	**0.746**	0.601	0.547
Homogeneity	0.134	0.180	**0.833**	0.118	0.128
Completeness	0.198	0.147	**0.559**	0.230	0.100
*Bacteria*					
Rand index	0.453	0.759	**0.823**	0.302	0.704
Homogeneity	0.146	0.618	**0.661**	0.074	0.247
Completeness	0.272	**0.713**	0.613	0.279	0.210
*Eukaryota*					
Rand index	0.450	0.688	0.729	0.248	**0.761**
Homogeneity	0.122	0.332	**0.380**	0.074	0.289
Completeness	0.295	**0.499**	0.483	0.413	0.277

The highest score for each row is written in bold

## Discussion

The evaluation framework, as it is presented in this work, can be considered a first step towards a larger benchmarking that would provide a standard in the field of comparing different methods that include pseudoknots. In this paper the selection of the dataset has been done with the task of reconstructing the phylogeny in mind. However, in general, a different selection can be made to evaluate a different choice of methods against other tasks, e. g., the classification of the known function or other known features of non-coding RNAs. The general approach of using dissimilarity measures and clustering to reconstruct homogeneous groups of molecules would remain the same. The only changes would be in the dataset and in the labels of the clusters. Other possible tasks to test using the framework could be, for instance, the classification of molecules having the same kind of pseudoknots or studying how different classes of pseudoknotted RNAs can be considered similar notwithstanding the different kind of pseudoknots they contain.

**Table 5:** Results of the evaluation framework applied to the three life domains (Archaea, Bacteria and Eukaryota) of 23S rRNA benchmark where single linkage was used in clustering

	Genus	PSMAlign	ASPRAlign	PskOrder	RAG-2D
*Archaea*					
Rand index	1.0	1.0	1.0	1.0	1.0
Homogeneity	1.0	1.0	1.0	1.0	1.0
Completeness	1.0	1.0	1.0	1.0	1.0
*Bacteria*					
Rand index	0.458	0.403	0.403	0.470	**0.584**
Homogeneity	0.242	0.155	0.155	**0.248**	0.216
Completeness	0.500	0.320	0.320	**0.510**	0.230
*Eukaryota*					
Rand index	0.776	0.829	**0.843**	0.695	0.800
Homogeneity	0.601	0.575	**0.676**	0.498	0.571
Completeness	0.551	0.604	**0.726**	0.548	0.655

The highest score for each row is written in bold

**Table 6:** Results of the evaluation framework applied to the three life domains (Archaea, Bacteria and Eukaryota) of 23S rRNA benchmark where complete linkage was used in clustering

	Genus	PSMAlign	ASPRAlign	PskOrder	RAG-2D
*Archaea*					
Rand index	1.0	1.0	1.0	1.0	1.0
Homogeneity	1.0	1.0	1.0	1.0	1.0
Completeness	1.0	1.0	1.0	1.0	1.0
*Bacteria*					
Rand index	0.487	**0.889**	0.828	0.473	0.591
Homogeneity	0.202	**0.858**	0.649	0.221	0.223
Completeness	0.307	**0.823**	0.697	0.387	0.220
*Eukaryota*					
Rand index	0.776	**0.857**	**0.857**	0.695	0.810
Homogeneity	0.601	0.650	**0.667**	0.445	0.536
Completeness	0.551	0.683	**0.689**	0.467	0.559

The highest score for each row is written in bold

**Table 7:** Results of the evaluation framework applied to the three life domains (Archaea, Bacteria and Eukaryota) of 23S rRNA benchmark where average linkage was used in clustering

	Genus	PSMAlign	ASPRAlign	PskOrder	RAG-2D
*Archaea*					
Rand index	1.0	1.0	1.0	1.0	1.0
Homogeneity	1.0	1.0	1.0	1.0	1.0
Completeness	1.0	1.0	1.0	1.0	1.0
*Bacteria*					
Rand index	0.487	0.633	**0.830**	0.473	0.591
Homogeneity	0.202	0.542	**0.653**	0.221	0.223
Completeness	0.307	0.718	**0.742**	0.387	0.220
*Eukaryota*					
Rand index	0.776	**0.857**	0.843	0.695	0.810
Homogeneity	0.601	0.650	**0.676**	0.445	0.536
Completeness	0.551	0.683	**0.726**	0.467	0.559

The highest score for each row is written in bold

Regarding the particular experiments presented in this paper, let us now discuss the values of the metrics obtained for the phylum reconstruction of 5S, 16S and 23S of Archaea, Bacteria and Eukaryota. We first observe that, in the case of comparisons based on Genus and PskOrder, as expected, the results are highly independent from the linkage method of clustering because the clusters that are computed are essentially the equivalence classes of the molecules with the same Genus or the same PskOrder. Moreover, in the case of Archaea 23S rRNAs all methods reach the maximum performance 1.0. This was also expected because in that case all the structures belong to organisms that have the same phylum in the ENA taxonomy. We retained these molecules for completeness as they were the only 23 S Archaea present in the CRW database from which we selected the benchmark.

**Table 8:** Results of the evaluation framework applied to the three life domains (Archaea, Bacteria and Eukaryota) of 5S rRNA benchmark where single linkage was used in clustering

	Genus	PSMAlign	ASPRAlign	PskOrder	RAG-2D
*Archaea*					
Rand index	0.631	**1.0**	0.48	0.631	0.677
Homogeneity	0.017	**1.0**	0.164	0.017	0.042
Completeness	0.052	**1.0**	0.130	0.052	0.075
*Bacteria*					
Rand index	0.423	**0.747**	0.553	0.423	0.423
Homogeneity	0.031	**0.557**	0.181	0.031	0.031
Completeness	0.079	**0.720**	0.463	0.079	0.079
*Eukaryota*					
Rand index	0.486	**0.569**	0.486	0.486	0.486
Homogeneity	0.093	**0.269**	0.093	0.093	0.093
Completeness	0.323	**0.725**	0.323	0.323	0.323

The highest score for each row is written in bold

**Table 9:** Results of the evaluation framework applied to the three life domains (Archaea, Bacteria and Eukaryota) of 5S rRNA benchmark where complete linkage was used in clustering

	Genus	PSMAlign	ASPRAlign	PskOrder	RAG-2D
*Archaea*					
Rand index	0.631	**1.0**	0.480	0.631	0.677
Homogeneity	0.017	**1.0**	0.164	0.017	0.042
Completeness	0.052	**1.0**	0.130	0.052	0.075
*Bacteria*					
Rand index	0.423	**0.930**	0.709	0.423	0.423
Homogeneity	0.031	**0.854**	0.455	0.031	0.031
Completeness	0.079	**0.816**	0.602	0.079	0.079
*Eukaryota*					
Rand index	0.486	**0.902**	0.486	0.486	0.486
Homogeneity	0.093	**0.784**	0.093	0.093	0.093
Completeness	0.323	**0.871**	0.323	0.323	0.323

The highest score for each row is written in bold

**Table 10:** Results of the evaluation framework applied to the three life domains (Archaea, Bacteria and Eukaryota) of 5S rRNA benchmark where average linkage was used in clustering

	Genus	PSMAlign	ASPRAlign	PskOrder	RAG-2D
*Archaea*					
Rand index	0.631	**1.0**	0.480	0.631	0.677
Homogeneity	0.017	**1.0**	0.164	0.017	0.042
Completeness	0.051	**1.0**	0.130	0.051	0.075
*Bacteria*					
Rand index	0.423	**0.830**	0.709	0.423	0.423
Homogeneity	0.031	**0.700**	0.455	0.031	0.309
Completeness	0.079	**0.784**	0.602	0.079	0.079
*Eukaryota*					
Rand index	0.485	**0.800**	0.485	0.485	0.485
Homogeneity	0.092	**0.576**	0.092	0.092	0.092
Completeness	0.323	**0.841**	0.323	0.323	0.323

The highest score for each row is written in bold

We also observe that the performances of Genus and PskOrder methods are frequently less than the others. This might be due to the high level of abstraction that these two methods apply on the information available in the secondary structures. Actually, as mentioned above, they essentially create equivalence classes. The other methods, ASPRAlign, PSMAlign and RAG-20, also make abstractions on the structures, but in a milder way: ASPRAlign neglects the primary sequence completely, but considers the whole structure of the molecule, PSMAlign considers also the shape, but not precisely (several bonds are excluded by the heuristic) and partially uses the sequence information, RAG-2D neglects the primary sequence as ASPRAlign, but does not consider the precise structure of the molecule making, instead, a topological-based evaluation of its complexity.

Let us now focus on the values obtained for 16S structures, which are the ones that are considered more conserved by evolution and are therefore the best candidates for taxonomy reconstruction. The performance of PSMAlign, ASPRAlign and RAG-2D can be considered similar in most cases, varying w.r.t. the different linkage methods and the metric used for the evaluation. Considering the three domains, in the case of Archaea, ASPRAlign always shows the best performance, while for Bacteria both PSMAlign and ASPRAlign provide the best performance, with the exception of single linkage parameter and random index metric in which RAG-2D performs better. It has to be noted, however, that the differences in the scores of these three methods are generally low. Similar consideration applies for Eukaryota, in which scores are similar and the best performance is reached by the three methods varying the linkage and the metric. However, taxonomy reconstruction for Eukaryota with the proposed methods seems to be slightly harder because the best performance scores in several cases are lower than the ones obtained for Archaea and Bacteria. These results might suggest that milder abstractions on the information contained in the secondary structures generally produce comparison methods that perform better, with a balance to be found in abstracting the primary sequence and the shape of the secondary structure. Moreover, we can say that the complexity of the organisms and the number of clusters to be reconstructed play an important role in the performance of the considered comparison methods. This suggests to continue the investigation by trying to reconstruct, surely for Eukaryota, but also for Bacteria, a taxonomy rank lower than phylum, i.e., class or order. This would permit to consider a lower number of molecules and could decrease the differences among them due to the reduction of the difficulty of the clustering when similar complex organisms are involved.

Let us now analyze the results of 23S structures. Here we can make considerations similar to the ones for 16S molecules, with two exceptions. First, the best performance scores between Eukaryota and Bacteria are very similar, so the complexity of the organism, for 23S, seems not to be a fundamental factor in taxonomy reconstruction. Second, in the case of Bacteria with single linkage parameter in clustering the best scores are shown by PskOrder and RAG-2D, while PSMAlign and ASPRAlign show lower scores. Genus has scores very similar to PskOrder. It is difficult to interpret these results, we can only say that a direction of investigation is to search for a relation between the single linkage parameter for clustering and a higher abstraction of the secondary structures.

Finally, let us consider the results on 5S structures. Being these structures pseudoknot-free the results show a significant difference w.r.t. the others. The first fact to notice is that PSMAlign always shows the best performance in the reconstruction of the phylum. This reflects the results presented by Chiu and Chen in [25], where PSMAlign was shown to outperform other comparison techniques on 16S *pseudoknot-free* secondary structures using the BRASERO framework [37]. Our results confirm this capability of PSMAlign that, in the case of Archaea, reconstructs the phylum exactly with score 1.0 for any parameter of the clustering. In the case of Bacteria and Eukaryota the best score is significantly greater than the other scores in all cases. We also note that Genus, PskOrder and RAG-2D in all cases show very similar or equal scores. This reflects the fact that pseudoknot-free structures do not have differences in their topology, therefore methods based on topology perform exactly in the same way. In the case of ASPRAlign, which is not based on the topology of the structures, but on a tree algebraic representation of their arc-diagram, there are better results, but worse than PSMAlign. This might be a consequence of the fact that ASPRAlign neglects the primary sequence completely, while PSMAlign considers it together with the relationships among the stems of the structure.

## Conclusions

We have proposed an evaluation framework for comparison methods of pseudoknotted RNA secondary structures. The framework performs the evaluation of a given comparison method by firstly using it to obtain a similarity/dissimilarity measure between any pair of molecules in a set of a given benchmark. Then, this measure is used by an agglomerative clustering algorithm to group the molecules into clusters that should correspond to groups having similar features. Finally, rand index, homogeneity and completeness metrics are used to evaluate how well the groups have been reconstructed.

To illustrate the framework we have defined a dataset of 16S, 23S and 5S rRNA molecules of Archaea, Bacteria and Eukaryota and we have used it to reconstruct the taxa of a selected taxonomy rank of a curated biological taxonomy. In particular we have reconstructed the phylum according to the ENA taxonomy. We have evaluated the comparison methods Genus, PSMAlign, ASPRAlign, PskOrder and RAG-2D for which the computed metrics have been reported and discussed.

As future work, we intend to use the evaluation framework, with the same dataset, to see if the considered comparison methods perform better on taxonomy ranks lower than phylum, especially for Bacteria and Eukaryota. Moreover we plan to extend the benchmark with other functional RNA families in order to continue the investigation on this phylogeny-based approach to evaluation. Different and new comparison methods, for instance [28], could also be included in our experiments. Finally, we want to use different curated taxonomies, e.g., SILVA [10, 11] and LTP [45], in order to compare the results with those obtained with ENA.

Another direction for future work is that of using the BRASERO [37] approach to confront the considered comparison methods with the ones for pseudoknot-free structures. Of course, in this case, we would use the BRASERO evaluation method and its benchmarks. As mentioned above this was done in [25] for PSMAlign and our results on pseudoknot-free 5 S structures confirm their results.

BRASERO uses an approach completely different from the one considered in this paper. In its case, to evaluate a comparison method, the benchmark consists of collections of molecules which are divided in three sets: reference set *R*, positive set *P* and negative set *N*. Molecules in sets *R* and *P* belong to the same family, while molecules in set *N* are chosen outside the considered family. Distances are computed between the molecules in *P* and *N* with molecules in *R* and the values are ordered. Then the molecules with the higher *n* values, where $n = |P|$, are classified as belonging to the family and the others as not belonging. A receiver operating characteristic (ROC) curve is then plotted to estimate the quality of the classification. A future project could be to define a framework that operates in this way for pseudoknotted structures.

Another important direction of future work is to use the framework to evaluate the capability of different methods to compare and classify the most probable secondary structures (with and without pseudoknots) relative to one specific sequence. This set can be defined using different folding methods, which may consider different parameters or different ranges of minimum free energy, on the same sequence. The folded secondary structures are not very stable and may refold or fold into different structures according to different environments. Therefore, the evaluation of the capability of different comparison methods of characterizing the probable secondary structures is certainly an interesting investigation.

A final interesting research direction is to consider other approaches to classify RNA secondary structures based on intersection graph or structural patterns [46–48] or take into account other abstractions like the one introduced in [49].

## Methods

### Methods for comparing RNA secondary structures with pseudoknots

In order to be included within our evaluation framework, a comparison method is required to handle pseudoknotted RNA secondary structures and to produce, for any given pair of molecules, a similarity/dissimilarity measure as a real number. More in detail, using the classification originally introduced by Evans in [50] and used by Blin and Touzet in [51], we assume that the comparison method is able to handle structures classified as NESTING or CROSSING, not falling in the UNLIMITED class. Note that this requirement does not limit the application of the framework in presence of multiple strands or complexes. In these cases the different components can simply be concatenated and considered as one.

We considered the available methods in the literature, described in “Background” Section, that satisfy the requirements above and for which a publicly available, currently working, software tool or website exists. Among these, we chose the five methods that are briefly described in the following for applying our evaluation framework. More precise details about each method can be found in the given references.

#### ASPRA distance

Algebraic ASPRA distance is a dissimilarity measure based on an algebraic representation of RNA secondary structures able to represent arbitrary pseudoknots using three operators: concatenation ($\odot$), nesting ($\Cap$) and crossing ($\bowtie$). The full algebraic definition of the operators can be found in [26]. Here, we briefly recall the main concepts. The operators are used to represent complex structures starting from single bonds (arcs in the arc-diagram). The concatenation operator permits to represent a structure that is followed by another one. Figure 1c shows a concatenation between two simple bonds. Nesting formalises the insertion of a structure into an arc (Fig. 1d shows the nesting of an arc into another arc), while the crossing operator models the crossing interactions among arcs, as illustrated in Fig. 1e.

The combination of these three operators permits to define structural RNA trees, which represent the structure of the molecule dropping the information about the primary sequence. For building the structural RNA tree of a structure, the idea is to select the arc having the rightmost paired nucleotide and to determine the relation (i.e, concatenation, nesting or crossing) between the considered arc with the rest of the structure. In the case of crossing, it is necessary to determine also the number of crossing that the arc performs with the remaining sub-structure. The left children of the root corresponds to the root of the sub-tree that represents the remaining structure. This process continues until the sub-structure becomes a single arc. The ASPRA distance is obtained by aligning the two structural RNA trees of the involved structures by using the classical tree alignment algorithm by Jiang et al. [52]. Let $S_1$ and $S_2$ be two RNA secondary structures and let $t_1$ and $t_2$ be their structural RNA trees. The ASPRA distance between $S_1$ and $S_2$, is defined as follows:

$$\begin{aligned} d_{aspra}(S_1, \ S_2) = \min \{\sigma _s(L) \mid L \text{ is } \text{ an } \text{ alignment } \text{ of } t_1 \text{ and } t_2 \} \end{aligned}$$

The scoring function $\sigma _s$ is defined on the nodes of the alignment tree of two structural RNA trees and, as usual, assigns different costs to the different edit operations: replacement, deletion and insertion.

The tool ASPRAlign [27] can be used to compute the ASPRA distance between two or more molecules and is freely available at https://github.com/bdslab/aspralign. To apply our framework we ran the tool on all pairs of structures in the benchmark and interpreted the obtained distance as a dissimilarity measure between them.

#### Progressive stem matching

In [25], Chiu and Chen faced the same problem of ASPRA distance, i.e., the development of a comparison method that would be efficiently applied to secondary structures with arbitrary pseudoknots. Also in their case, the key point was the high conservation of structures w.r.t. sequences so they could focus more on aligning the structural part of the molecules. The idea was that of considering conserved stem patterns, i.e., subsets of the bonds that define a stem and their structural relationships: concatenation (called parallel), nesting and crossing (called pseudoknot). These stems and their relationships are encoded in a stem graph and then the minimum cost error-correcting graph matching (mcECGM) [53] is computed between the two graphs of a given pair of structures. However, since ECGM is in general NP-complete, the authors created an heuristic, called Progressive Stem Matching (PSM), to reduce the number of vertices in the graphs to be considered. In this way they could computationally treat large RNA molecules such as 16S and 23S rRNAs.

This approach was implemented in the tool Progressive Stem Matching Alignment (PSMAlign) available at http://homepage.cs.latrobe.edu.au/ypchen/psmalign/. In [25], the ability of PSMAlign to identify similar structures was measured using BRASERO [37] on 16S rRNAs and signal recognition pattern datasets. PSMAlign was compared with RNAforester [3, 13], MiGaL [15] and Gardenia [17]. The authors showed that PSMAlign over-performed the other tools. However, since such tools do not accept structures with pseudoknots the comparison could only be done on pseudoknot-free structures. For testing pseudoknotted structures, in absence of a dedicated benchmark, the authors randomly selected some 23S RNAs molecules and compared the times for computing the alignments using PSMAlign and the times for computing plain classical sequence alignment using the Needelman-Wunsh algorithm [54].

In order to apply our framework we ran PSMAlign on all the pairs of structures in our benchmark and collected the alignment costs. We interpreted such costs as a dissimilarity measures between the input structures.

#### Genus topological invariant

The genus of an RNA secondary structure is a measure of its complexity. It can be defined as the minimum number of handles that we need to attach to a sphere to get a surface where the arc-diagram of the structure can be drawn without any crossing, still preserving the property that all the arcs are attached to the backbone from the same side, like in the plane. An arc-diagram that does not present any crossing can be drawn on a sphere with no handles.

Full details on how the genus of a structure is defined and computed can be found in [19, 20, 55]. The Genus for Biomolecules database [56, 57] at https://genus.fuw.edu.pl permits to compute the genus trace plot of a molecule. We defined a Python script implementing the algorithm presented in [58], which is able to compute the genus of a structure. The script is available at the https://github.com/bdslab/TaxonClassifier repository and was used to determine the genus of all the molecules of the benchmark. Since the genus is just a feature of a molecule, then molecules with the same genus can be considered belonging to the same equivalence class (or group). Thus, the taxa that can be computed by this method are essentially these equivalent classes. However, to apply our framework we had to formally satisfy the requirement of having a similarity/dissimilarity measure. We simply defined a trivial dissimilarity between two different structures as the absolute value of the difference of their genera.

#### Pseudoknot order

The pseudoknot order, introduced in [29], is a measure to quantify the structural complexity of a secondary structure with pseudoknots. It is defined as the minimum number of regions that should be eliminated from a structure in order to get a pseudoknot-free one. Here a region is a set of nested pairs in which there are not unpaired nucleotides, that is stacks of helices with no bulges or inner loops. Every region is assigned an order, which is 0 if it does not cross with any other region and greater than 0 if it forms a crossing. The regions assigned with order 1 are those that cross with the ones of order 0. Then the regions assigned with order 2 are those that cross with the ones of orders 0 and 1 previously determined. The process continues until all the regions are assigned with an order. The higher order that is found is considered the pseudoknot order of the whole structure.

In [31], the authors showed that the pseudoknot order is useful in understanding the hierarchy of RNA folding. In particular, it is assumed that pairs with order 0, the pseudoknot-free ones, are formed first. Then, bonds of order 1 are formed, followed by the ones of order 2 and so on.

The algorithms to compute the orders have improved over the years. In [30], graph-based algorithms are proposed, among which the MILP (mixed-integer linear programming) one, which is shown to be the most performant in terms of quality of the computed solutions. We used the MILP approach on all the molecules of the benchmark to determine their pseudoknot order. Similarly to the genus topological invariant, in order to fit in our evaluation framework, we defined a dissimilarity measure as the absolute value of the difference of the pseudoknot orders of two molecule.

#### RAG-2D: RNA As Graph

Graph theory has been used to represent RNA secondary structures in various scenarios [59, 60]. In the last two decades, Schlick and co-workers have used *dual graphs* to formalize RNA secondary structures with pseudoknots [33–35] within the RNA As Graph approach (RAG-2D—http://www.biomath.nyu.edu/?q=rag/home). To convert an RNA secondary structure into its dual graph, RNA structural elements—i.e., helix or stem, bulges, hairpin loops, internal loops, and multi-loops—and their relations are considered as follows:

- Each RNA stem is represented as a vertex of the graph;
- An edge represents any single strand that has more than one unpaired nucleotide and occurs in segments connecting structural elements;
- The $3'$ and $5'$ ends do not have any representation.

Dual graphs describe the connectivity without specifying the geometric aspects of the secondary structure. Moreover, they do not specify the exact sequence or the length of the considered RNA molecule.

The connectivity of a graph is described by its Laplacian matrix. The Laplacian matrix is defined as the difference between the degree matrix *D* and the adjacency matrix *A*. The degree matrix *D* is an $n \times n$ diagonal matrix with diagonal entries $d_{ii}$ equal to the number of edges incident on vertex *i*. The adjacency matrix *A* for a dual graph has entries $a_{ij}$ equal to the number of edges between vertex *i* and *j*, and $a_{ii} = 2$ if there is a self-loop on vertex *i*. By construction, the Laplacian matrix is positive semidefinite with $\lambda _1 = 0$ as its smallest eigenvalue and associated eigenvector $\mu _1 = (1,1, \dots ,1)^T$. Since the graphs that are considered are connected, the second smallest eigenvalue of *L*, the Fiedler value $\lambda _2$, is positive and increases with the compactness of the graph. The Fiedler value describes the algebraic connectivity of a graph. However, using it alone is insufficient to distinguish graphs. As proposed in [36], to develop characteristics that better reflect the graph topology, two features, *s* and *e*, are defined as follows:

1. Calculate the normalized Fiedler vector $\mu _2 = (\mu _{2,1}, \mu _{2,2}, \dots , \mu _{2,n})^T$ of the Laplacian matrix *L*;
2. Sort the Fiedler vector components $\{\mu _{2,i}\}_{i=1}^n$ in ascending order and denote the ordered components $\{v_i\}_{i=1}^n$;
3. Scale each $v_i$ to be $\tilde{v}_i = \frac{{v}_i(n-1)}{v_n -v_i}$;
4. Perform linear regression on the points $(1, \tilde{v}_1), (2, \tilde{v}_2), \dots , (n, \tilde{v}_n)$ to obtain slope *s* and mean squared error *e*.

We computed the dual graphs of all the molecules of the benchmark and the corresponding *s* and *e* features. Then these two features were used to define a distance between two molecules by considering the Euclidean distance.

### Agglomerative clustering

Agglomerative clustering is a hierarchical clustering method of cluster analysis based on a bottom-up approach. It starts taking singleton clusters that contain only one data object per cluster at the bottom level. It continues merging pairs of clusters at a time to build the hierarchy of the groups [61]. Such hierarchy can be interpreted using the standard binary tree terminology. The root or level 0 represents all data and forms the apex, and each other level corresponds to some set of clusters. The child entries (i.e., nodes) correspond to the clusters and identify subsets of the entire dataset. The group elements correspond to the leaves (i.e., singleton points) of the sub-tree having the current node as root. This cluster hierarchy is called a dendrogram, the same structure used in phylogenetics for phylogenetic trees. The main advantage of using a hierarchical clustering method is that it allows for cutting the hierarchy at any given level and obtaining the clusters correspondingly. This feature makes it a suitable clustering method in automatic taxonomy construction.

More in detail, the algorithm consists of the steps reported in Algorithm 1. First, a dissimilarity matrix is constructed, either given as input, e.g. as a distance matrix, or computed from a proximity/similarity measure. All the data points are then assigned to their own singleton cluster. At each iteration the closest clusters pairs are merged and the dissimilarity matrix is updated correspondingly. This process continues until the final maximal cluster that contains all data is achieved. Various clustering schemes share this general procedure, but they differ in the way in which the measure of inter-cluster dissimilarity is updated after each step. The most common methods are: single, complete, average, and ward linkage. Single link clustering computes the similarity of the two groups as the similarity between their most similar (nearest neighbor) members. Complete link clustering measures the similarity of two clusters as the similarity of their most dissimilar members. Average linkage determines the distance between two clusters as the average distance between all pairs of individuals from each group. Ward linkage measures the similarity of two clusters as the sum of squared differences within all clusters. However, ward linkage can be applied only when the dissimilarity matrix contains Euclidean distances, which is typically not the case in the values computed by comparison methods. 
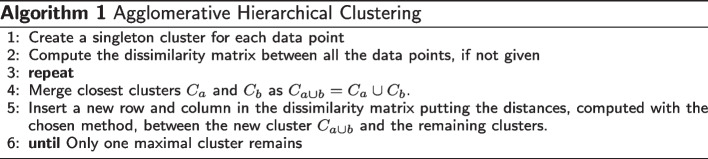


### Metrics for clustering evaluation

Evaluating the performance of a clustering algorithm is not trivial. Evaluation metrics should be used that define separations of the data similar to some ground truth set of classes. Moreover, such metrics should satisfy some assumptions such that members of the same group are more similar than members of different clusters according to some similarity metric. In our work, we use three metrics: rand index [40], homogeneity and completeness [41]. They will be used on two assignments, i.e., the sets of labels classified by the clustering algorithm and the sets of labels present in the chosen curated taxonomy. The obtained number of each metric is in the interval [0, 1] and gives the performance of the method. 1 is the maximal performance, i.e., the computed clusters coincide with the known ones.

#### Rand index

A rand index *R* is a function that measures the similarity of two assignments. It computes the measure between two clusters by considering all pairs of samples and counting pairs that are assigned in the same or different clusters in the predicted and the knowledge of the ground truth class assignments. Let $S = \{e_1, e_2, \ldots , e_n \}$ be a set of elements that are clusterised and let *X*, *Y* be two clusterings of *S*. Let *a* be the number of pairs of elements in *S* that are in the same subset in *X* and in the same subset in *Y*. Let *b* be the number of pairs of elements in *S* that are in different subsets in *X* and different subsets in *Y*. Let *c* be the number of pairs of elements in *S* that are in the same subset in *X* and different subsets in *Y*. Let *d* be the number of pairs of elements in *S* that are in different subsets in *X* and the same subset in *Y*. The rand index is defined as

$$\begin{aligned} R = \frac{a+b}{a+b+c+d} \end{aligned}$$

#### Homogeneity

The homogeneity score, *h*, measures how much the elements in a clustering are similar. The score is defined using Shannon’s entropy

$$\begin{aligned} h = 1 - \frac{H(C|K)}{H(C)} \end{aligned}$$

where *H*(*C*|*K*) and *H*(*C*) are the conditional entropy of the classes given the cluster assignments and the entropy of the classes, respectively. Formally,

$$\begin{aligned} H(C|K)= & {} - \sum _{c=1}^{|C|} \sum _{k=1}^{|K|} \frac{n_{c,k}}{n} \cdot \log \left( \frac{n_{c,k}}{n_k}\right) \\ H(C)= & {} - \sum _{c=1}^{|C|} \frac{n_{c}}{n} \cdot \log \left( \frac{n_{c}}{n}\right) \end{aligned}$$

where *n* is the total number of samples, $n_c$ and $n_k$ are the number of samples respectively belonging to class *c* and cluster *k* and where $n_{c,k}$ is the number of samples from class *c* assigned to cluster *k*.

#### Completeness

The completeness score, *c*, measures how much similar elements are put together. Like to the homogeneity score, it is defined in terms of Shannon’s entropy

$$\begin{aligned} c = 1 - \frac{H(K|C)}{H(K)} \end{aligned}$$

where *H*(*K*|*C*) and *H*(*K*) are conditional entropy of clusters given the class and the entropy of clusters, respectively.

## Data Availability

The RNA secondary structures of Archaea 16S, Archaea 23S, Archaea 5S, Bacteria 16S, Bacteria 23S, Bacteria 5S, Eukaryota 16S, Eukaryota 23S and Eukaryota 5S rRNAs are available in different formats at https://doi.org/10.6084/m9.figshare.20731783.v1 under the CC-BY 4.0 License. The list of molecules in each set with their ENA taxonomy information are given in the spreadsheets Archaea.xlsx, Bacteria.xslx and Eukaryota.xslx, which are included in the same repository. For each molecule in these files the following information is given: Progressive Number. rRNA—the family of the molecule. Organisms—the organism’s name to which the molecule belongs. The accession number of the molecule can be found in the headers of the relative bpseq and ct files. Benchmark ID—unique ID of the molecule in the benchmark. Phylum—phylum taxon associated to the molecule according to the ENA taxonomy. Class—class taxon associated to the molecule according to the ENA taxonomy. Order—order taxon associated to the molecule according to the ENA taxonomy. The repository https://github.com/bdslab/TaxonClassifier contains: the two Python scripts to execute the evaluation framework; all the scripts that were used to compute the distances/features of the considered sets of molecules and the considered comparison methods; all the input files that can be given to the framework to reproduce the results presented in this paper, namely the CSV files containing the list of molecules for each set with the ENA phylum information and the CSV files containing the computed distances (or pairs of features) for each set; the results presented in this paper as output of the framework scripts.

## Abbreviations

rRNA: Ribosomal RNA
ASPRA distance: Algebraic structural pseudoknot RNA alignment distance
ENA: European Nucletoide Archive
PSM: Progressive stem matching
PskOrder: Pseudoknot order
RAG-2D: RNA As Graph 2D
